# Description of vertebral and liver alveolar echinococcosis cases in Cynomolgus monkeys (*Macaca fascicularis*)

**DOI:** 10.1186/s12917-015-0520-8

**Published:** 2015-08-12

**Authors:** Julie Brunet, Pierrick Regnard, Bernard Pesson, Ahmed Abou-Bacar, Marcela Sabou, Alexander W. Pfaff, Ermanno Candolfi

**Affiliations:** Institut de Parasitologie et Pathologie Tropicale, EA 7292, Fédération de Médecine Translationelle, Université de Strasbourg, 3 rue Koeberlé, 67000 Strasbourg, France; Centre de Primatologie UdS - SILABE (Simian Laboratory Europe) ADUEIS, Fort Foch, 67207 Niederhausbergen, France

## Abstract

**Background:**

*Echinococcus multilocularis*, the causative agent of alveolar echinococcosis, is a fox tapeworm widely distributed in Europe with an increase of endemic area in recent years. Many mammal species including humans and non-human primates can be infected by accidental ingestion of eggs.

**Case presentation:**

In March 2011, a 5-year-old zoo-raised male cynomolgus macaque (*Macaca fascicularis*) presented a paresis of the lower limbs which evolved into paralysis. Lesions in liver and vertebra were observed on tomography scan. *E. multilocularis* infection was diagnosed post-mortem by morphological and histological examination and detection of Em DNA by polymerase chain reaction. Serodiagnosis of other primates of the colony using enzyme-linked immunosorbent assay (ELISA) was negative. In June 2013, at necroscopy, a hepatic and a paravertebral masses were detected in a second cynomolgus macaque of the same colony. Serology and DNA isolated from hepatic and abdominal cysts confirmed *E. multilocularis* infection.

**Conclusions:**

We described hear vertebral and liver localization of alveolar echinococcosis in non-human primates. The animals lived in an indoor/outdoor housing facility, where the probable mode of contamination is by ingestion of food foraging around the enclosure which could be contaminated with fox feces. Serological survey in the facility should allow us to estimate the risk of human contamination and the zoonotic risk of monkey infection due to environmental contamination.

## Background

Alveolar echinococcosis (AE) is a zoonotic infection caused by the larval stage of *Echinococcus multilocularis* (*E. multilocularis*)*.* In Europe, the adult stage of this cestode is found mostly in the digestive tract of red fox (*Vulpes vulpes*). Rodents are the main intermediate hosts where the parasite develops into the metacestode larva mainly in the liver as multivesiculated cysts [1]. *E. multilocularis* prevalence has increased in the European fox population in the past decade associated with a westward spread and higher prevalence in France. Parasite prevalence in foxes in Alsace region was at 4.3 % between 1983 and 1988 (327 foxes collected), and estimated at 29 % in the North Alsatian Bas Rhin department between 2005 and 2010 (7 foxes collected) [2, 3]. While foxes are involved in a wild animal cycle life cycle of the parasite, domestic carnivores including cats and dogs could be potential definitive hosts involved in human infection [1]. In France, the prevalence in canine population is lower than 1 % [4]. Like humans, non-human primates (NHP) have been reported to be accidental intermediate hosts of this parasite. *E. multilocularis* infections have been described in macaques but also in gorillas and orangutans in Europe or in Japan by accidental ingestion of fox feces containing eggs or by ingestion of contaminated food. In NHP, AE develops primarily in liver and in some cases in other organs. In a Swiss study on captive monkeys, the liver was affected in 100 % of the AE cases, the lung in 40 %, abdominal cavity in 33 % and lymph nodes in 13 % [1]. In endemic regions, captive monkeys are considered as a population at risk [5–10]. Cynomolgus monkeys (*Macaca fascicularis*) are known to have the highest risk of infection and to be very susceptible monkey species. 33.3 % of the *M. fascicularis* were found to be infected in the German Primate Center [1, 10]. A number of AE cases have been described in captive cynomolgus monkeys in Switzerland where AE involved mainly the liver, but also the pancreas, lungs, kidneys or lymph nodes. Protoscolices were frequently found in lesions, contrary to humans where the germinal membrane of multilocular cysts usually does not produce protoscolices [7, 8, 11]. Diagnosis of AE is often made post-mortem as symptoms may only appear in advanced stages of the disease. However, regular serology screening of primate colony has been shown to be a useful method to identify monkeys at risk in regions where *E. multilocularis* is endemic [12].

We report here two cases of AE infection involving the liver and vertebrae in cynomolgus monkeys in captivity in Strasbourg, France. AE infection has been previously reported in primates in France, but for the first time here in *Macaca fascicularis* [13]. Moreover, most of the previous cases report cystic lesion of the liver. This is, to our knowledge, the first report of an AE with bone localization in NHP.

## Case presentation

**Fig. 1:**
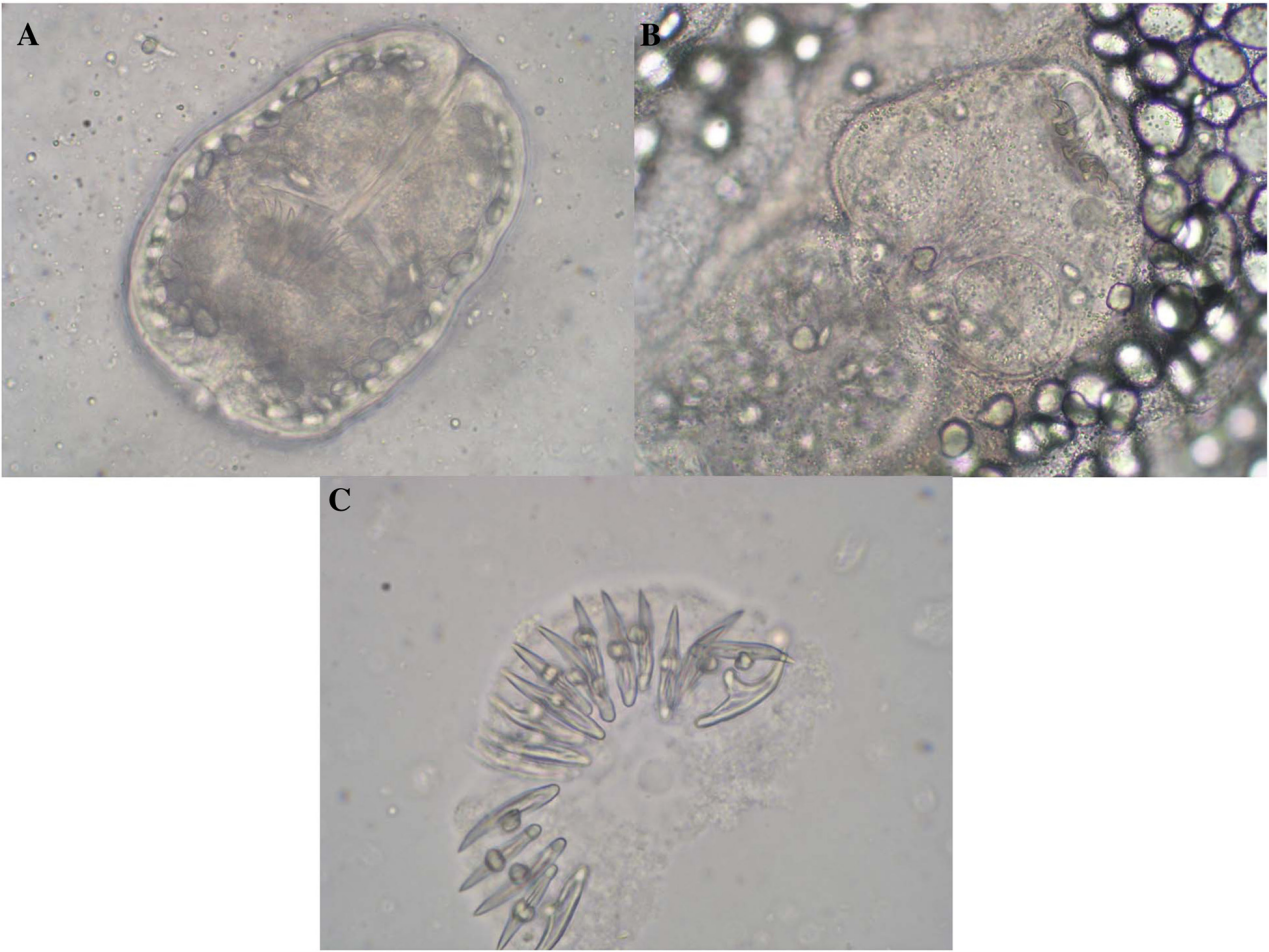
Details of the protoscolices invaginated (**a**) or evaginated (**b**). Morphology of rostellar hooks of *E. multilocularis* from the first case (**c**)

The first animal was a 5-year-old male cynomolgus macaque (*Macaca fascicularis*) born in Mauritius and raised for 3 years in a group of 11 monkeys in an indoor/outdoor facility with open-air enclosure at SILABE platform, located near the Niederhausbergen forest (Alsace, northeastern France). Its diet had consisted of pellets completed with fruits and seeds. During the 3 years, 3 sanitary checks were performed, including general check-ups, weighing, tuberculination and injections of ivermectine (1 mg/3 kg, subcutaneously). No abnormalities were reported for the concerned animal until March 2011 when it presented a paresis of the lower limbs which evolved into paralysis. Blood tests revealed increased monocyte (2302/mm^3^; reference: 810/mm^3^) and neutrophile counts (11,040 /mm^3^; reference range: 5000-7000/mm^3^). Other values were within normal range. A computed tomography scan revealed the presence of one large hepatic mass (5 cm). A second one (3 cm), located in paravertebral region, was associated with vertebral lesions (T9-T11) and spinal deformation. The monkey was euthanatized and submitted to postmortem examination. Multiple vesicles and alveolar cystic lesions characteristic of AE were found (1–3 mm). Necrosis was observed in the liver parenchyma and cysts were surrounded by eosinophile and neutrophile infiltration. No cyst formation was present in the other tissues examined (lung, pancreas or spleen). Vesicles contained multiple protoscolices and calcareous corpuscles. Protoscolices were both invaginated or evaginated (Fig. 1a and b). Hooks were visible (<30 μm) (Fig. 1c). Serology was highly positive for *Echinococcus multilocularis* (using enzyme-linked immunosorbent assay (ELISA) with Em2+ antigen, Bordier Affinity Products SA, Crissier, Switzerland) and completely negative for *Echinococcus granulosus*. DNA isolated from the hepatic cysts confirmed the identification of *E. multilocularis* (200 bp), using primer pair EM-H15/EM-H17 and cycling conditions as described by Georges et al. [14]. In April 2011, all animals of the colony were screened for *E. multilocularis* antibodies by serology (ELISA). All monkeys were asymptomatic and seronegative.

Twenty-seven months later, during a routine check-up, an abdominal enlargement was observed in a second cynomolgus macaque of the colony. At necropsy, a hepatic mass (12 cm) and a paravertebral mass (2 cm) close to the spine were observed associated with atrophy of the lungs and an important volume of ascites. The animal was an 11-year-old male cynomolgus macaque born in Mauritius that had lived in the center for 7 years. Half of the liver parenchyma was destroyed and multilocular cysts were present in both masses and were consistent with infection by the metacestode stage of *E. multilocularis*. Vesicles contained multiple protoscolices. Serology and DNA isolated from hepatic and abdominal cysts confirmed *E. multilocularis* infection. In September 2013, all animals of the colony were screened again for *E. multilocularis* antibodies by serology and all monkeys were seronegative.

## Discussion

NHP deaths due to *E. multilocularis* infection have been already reported in a number of zoos to date. However, these were the first cases of AE reported in macaques in France enclosures. *M. fascicularis* seem to be much more susceptible to *E. multilocularis* than humans. Indeed, contrary to humans or gorillas, protoscolices are produced, accompanied by rapid proliferation and non extensive necrosis or fibrosis [1]. In humans, the primary infection site is the liver for almost all of the patients. Extrahepatic lesions are frequently found in diaphragm, kidney, adrenal glands, lungs, pleura and brain. Bone localizations were already described in our region in two patients, including one lethal [14]. That site for *E. multilocularis* larvae is exceptional, occurring in 1 % of all cases [15, 16]. Previous cases of *E. multilocularis* infection in macaques described liver and abdominal cavity, kidney or lung lesions [1]. The peculiarity of our cases is the simultaneous involvement of the liver, vertebrae and spinal cord, observed for the first time in NHP.

Since Mauritius is not a known *E. multilocularis* endemic area, contamination was acquired in France. Oral ingestion of the eggs with infested food is highly suspected. Macaques are sometime seen foraging grass taken from nearby lawns possibly contaminated by fox feces. Foxes are noticed around the enclosure and are responsible for most of the environmental dissemination and contamination with *E. multilocularis* eggs [1]. *E. multilocularis* eggs can survive and remain infective in the environment for months [17]. The reported prevalence of *E. multilocularis* among foxes in Alsace was 4.3 %, but may be considerably higher now [2, 3]. The risk of monkey infection of the SILABE platform with *E. multilocularis* is real as shown by the recent description of a macaque infection by *Taenia martis* tapeworm probably acquired through egg ingestion directly from carnivore feces or during foraging activities [18].

These cases reflect the likely contamination of the environment. The Centre of Primatology is located in a forest region of eastern France where *E. multilocularis* is endemic. The second infected monkey, while initially negative, showed AE 27 months later, reflecting the possibility of a rapid disease development and the necessity for regular screening of the colonies in endemic regions as it was previously described by Rehmann et al. [12].

Although high prevalence of *E. multilocularis* in wild and domestic animals is not always associated with high prevalence of AE in humans, environmental contamination accentuates the risk of infection among captive monkeys but also the potential risk for the Center staff [19]. An analysis of the prevalence of *E. multilocularis* in the monkey population of the Centre of Primatology and therefore the risk factors associated with human infection is essential due to the severity of the disease in humans. As monkey groups are raised in semi-free ranging condition or in indoor/outdoor facilities, preventive measures against carnivores roaming around the park and limiting foraging activities are necessary.

## Conclusions

To our knowledge, this is the first description of *E. multilocularis* infection in NHP associated with liver and vertebra lesions. These cases confirm the presence of *E. multilocularis* in the areas around the SILABE platform and direct action toward the parasite must be considered.

## Abbreviations

ELISA: Enzyme-linked immunosorbent assay
AE: Alveolar echinococcosis
NHP: Non-human primates
